# Urinary Biopyrrin Levels and Their Relationship with the Menstrual Cycle and Concomitant Symptoms Among Healthy Nonpregnant Women of Reproductive Age: A Cohort Study

**DOI:** 10.1089/whr.2023.0074

**Published:** 2023-12-28

**Authors:** Yoko Chiba, Risako Hayashi, Hidehiro Hayashi, Ting-Fang Kuo, Wataru Hojo, Takuya Iwabuchi

**Affiliations:** ^1^ Department of Nursing, Kyoto College of Nursing, Kyoto, Japan.; ^2^ Cellspect Co., Ltd., Iwate, Japan.

**Keywords:** urinary biopyrrin, oxidative stress, healthy non-pregnant women, menstrual cycle, representative value

## Abstract

**Background:**

Urinary biopyrrin (UBP) is an oxidative metabolite formed from the reaction of bilirubin with reactive oxygen species. Previous studies have explored the relationship between UBP levels and certain diseases or pregnancy. However, UBP levels in healthy nonpregnant women have not been well examined. We aimed to clarify the representative value of UBP in healthy nonpregnant women and explore its relationship with menstrual cycles and concomitant symptoms.

**Methods:**

We included healthy, nonpregnant Japanese women aged 20–39 years with normal body mass index and menstrual cycle. In total, 1260 urine samples collected during 43 menstrual cycles of 36 women were analyzed to determine the representative values and reference intervals of UBP levels. The correlation between daily UBP levels and the order of the day was explored, and median UBP levels of 5-day clusters were compared using Friedman and Mann–Whitney *U* tests. These analyses were also conducted in women with concomitant symptoms during the menstrual cycle.

**Results:**

The median UBP level in all samples was 0.2291 (reference: 0.0102–2.9335) μmol/gCr. There was no significant relationship between the median UBP level and menstrual cycle, regardless of the presence of self-manageable symptoms during or before menstruation.

**Conclusions:**

The representative UBP value and its reference interval can serve as standards for comparison with other populations. Our findings suggest that the UBP level may be an objective oxidative stress indicator that is less sensitive to menstrual cycle and concomitant symptoms. UBP levels in healthy nonpregnant women could be assessed regardless of the menstrual cycle and concomitant symptoms.

## Introduction

In a society with a declining birth rate, such as Japan, women's preconception health is becoming more important. With the constantly increasing female employment rate in Japan (22–44 years of age), which was 79.8% in 2022,^1^ more women now have a job while having to bear the pressure of reproduction along with social responsibilities. Among women of reproductive age, depression is one of the 10 indicators to evaluate their preconception health.^2^ Stress, anxiety, depression, and other mental health conditions are common among these women,^3^ and therefore, have adverse effects on their child or children and family.^4^

In response, stress management among women of reproductive age is of utmost concern in public health. For mental health assessment, subjective indices, such as Profile of Mood States Questionnaire, Edinburgh Postpartum Depression Scale, and General Health Questionnaire (GHQ) scores, as well as objective indices, such as stress-related hormones or biomolecules, are often used.

Biopyrrin is an essential antioxidant and an oxidative metabolite of bilirubin. Bilirubin is biosynthesized from heme through the activities of heme oxygenase and biliverdin reductase and has previously been regarded as a potentially toxic waste product of heme catabolism.^5^ However, recent studies have revealed that bilirubin is a physiological antioxidant that scavenges oxygen and peroxyl radicals *in vitro* and *in vivo*.

Bilirubin reacts with reactive oxygen species to form hydrophilic oxidative metabolites that are excreted in urine.^5^ Shimoharada et al. identified the oxidative metabolites of bilirubin in human urine, now collectively called biopyrrin.^6^ Recently, high urinary biopyrrin (UBP) levels have been reported in patients with acute myocardial infarction, atrial fibrillation, idiopathic Parkinson's disease, atopic dermatitis, and chronic schizophrenia.^7–11^

Furthermore, some studies have suggested that psychotic status is associated with increased levels of oxidative metabolites of bilirubin in human urine, such as in schizophrenia and depression,^12^ and even with stress in making a speech at a conference.^13^

Regarding women of reproductive age, some studies have investigated UBP levels during pregnancy. Matsuzaki et al.^14^ revealed that UBP levels were higher in pregnant women than in nonpregnant women. Moreover, UBP levels were significantly higher in the third trimester of pregnancy than in the first and second trimesters. These results are consistent with the finding of Tateoka and Takahashi,^15^ where cortisol concentration, a stress indicator, increased significantly in early and mid-pregnancy and even more in late pregnancy.

In addition, Matsuzaki et al.^14^ found that elevated UBP levels were related to increased symptoms of pregnancy-induced hypertension; such as hypertension and urinary protein, and increased 3-hydroxy butyric acid, glucose levels and GHQ scores, as well as decreased high-density lipoprotein and fatty acid levels. However, the same study demonstrated no significant relationship between UBP levels and daily activities, such as eating, working, and sleeping, among pregnant women.^14^

The UBP level in healthy nonpregnant women has not been well examined, although it could be a standard value for comparison with those who are pregnant or sick. Matsuzaki et al.^14^ collected 35 urine samples from healthy nonpregnant women as a control group, and the mean UBP level (with standard deviation) was 1.7 (0.9) μmol/gCr. Tada et al.^16^ showed that the mean UBP level among 40 healthy volunteers (including five males aged 20–23 years with body mass index [BMI] <25.0 kg/m^2^) was 1.21 (0.61) U/gCr.

Nevertheless, in both studies, it was unclear when the urine samples were collected during the menstrual cycle. In addition, Tada et al.^16^ compared UBP levels during menstrual and nonmenstrual periods and showed that menstruation did not affect UBP levels; however, this analysis only included four women. Therefore, our study aimed to clarify the standard UBP values among healthy nonpregnant women and explore the relationship of UBP with the menstrual cycle and concomitant symptoms to clarify the appropriate sampling period.

## Materials and Methods

### Study design, setting, and participants

This prospective cohort study was conducted in Japan between January 2018 and March 2020. Nonpregnant Japanese women without specific diseases, aged between 20 and 39 years, with normal BMI (18.5 ≤ BMI <25.0 kg/m^2^), and who had a normal menstrual cycle (25–38 days^17^) were invited to this study through convenience sampling. Only those who could manage their symptoms during and before menstruation independently (without gynecological treatment) were included. Those receiving oral contraceptives at the time of recruitment were excluded.

One of the research assistants provided a detailed explanation of this study to all interested women using a written form, and everyone decided to participate at their own will. Written informed consent was obtained from each one before participation.

### Collection of personal and menstrual information

Age, weight, and height were confirmed, and BMI was calculated. The menstrual cycle (the number of days in one cycle) on usual days and self-manageable symptoms during and before menstruation were also recorded.

### Urine sample collection and biomarker measurement

Participants were asked to collect urine samples every morning during one menstrual cycle. One cycle was defined as the second day of menstruation to the first day of the following cycle. After successful participation in their first cycle, some women willingly continued to collect urine samples during their second cycle.

Urine samples were immediately placed in shaded bags to avoid light exposure. The samples were wrapped in an additional plastic bag and stored in a home freezer or cooler bag, according to the convenience of the participants, before collection by the research assistant. The collected samples were quickly sent to an analysis center through a courier service under freezing conditions.

The urine samples were analyzed within 3 months to check UBP and creatinine levels, centrifuging each sample at 3000 × *g* before measurement. UBP levels were measured in duplicates using a Biopyrrin ELISA Kit (^®^Cellspect, Iwate, Japan). The results were corrected for urinary creatinine concentration, which was determined using an automated bioanalyzer (Hitachi, Tokyo) and Iatro LQ CRE (^®^LSI Medience, Tokyo).

### Analyses

Age, weight, BMI, menstrual cycle, and self-manageable symptoms during and before menstruation were analyzed descriptively to clarify characteristics of participants and their menstrual cycles. Representative values and reference intervals of the UBP levels were calculated based on a normality test of UBP levels for all samples.

To observe the trend in UBP levels in the follicular phase, correlations between daily UBP levels from the second day to the 14th day of menstruation and the order of days were confirmed. Similarly, in the luteal phase, correlations between daily UBP levels during the 14 days from the last day of menstruation and the reverse order of days were confirmed. The same analyses were conducted only for cycles where participants reported self-manageable symptoms during or before menstruation.

To explore whether UBP levels during menstruation and ovulation differed from those on other days, the median UBP levels among each group were compared every 5 days from the second day of menstruation. To confirm whether the UBP levels before menstruation differed from those on other days, the median UBP levels among each 5-day cluster from the last day of menstruation in reverse order were also compared.

These analyses were implemented only for cycles where participants reported self-manageable symptoms during or before menstruation. Additionally, median UBP levels in every 5-day cluster with and without self-manageable symptoms during or before menstruation were also compared.

The level of significance was set at *p* < 0.05. The SPSS Statistics 26.0 software package (^®^IBM SPSS, Washington, IL, USA) was used for statistical analysis.

### Ethical considerations

The study protocol was approved by the Ethics Committee of Kyoto College of Nursing, Kyoto, Japan (approval number: 201710-2, as of October 31, 2017). All research assistants were registered midwives certified in Japan because menstruation and home visits for urine sample collection are sensitive issues for women. All samples were treated anonymously.

## Results

### Number of participants and menstrual cycles

**FIG. 1.**
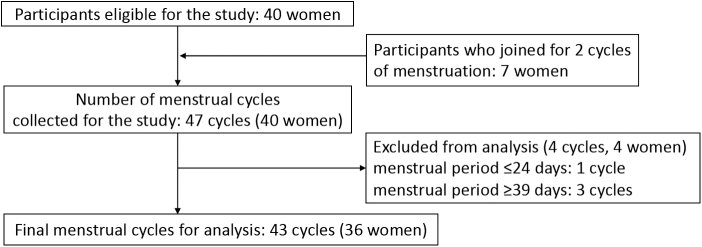
Number of participants and menstrual cycles for analysis.

Forty eligible women were enrolled in this study (Fig. 1). Seven women agreed to undergo urine sampling during two menstrual cycles; thus, samples were collected during 47 menstrual cycles. However, samples collected during four cycles of four women were excluded because the actual length of their menstrual cycle was either too short (≤24 days; *n* = 1) or too long (≥39 days; *n* = 3). Finally, urine samples collected during 43 menstrual cycles of 36 women (a total of 1260 samples) were analyzed.

### Characteristics of participants and their menstrual cycles

The mean age of participants was 27.7 (4.9) years, mean weight was 52.6 (4.6) kg, and mean BMI was 20.7 (1.3) kg/m^2^ (Table 1). The reported and actual mean numbers of menstrual cycles were 29.6 (2.3) and 30.0 (3.3), respectively. Approximately 60% of the participants had symptoms during menstruation, while slightly <50% had symptoms before menstruation, all of which were self-manageable.

**Table 1.** Characteristics of the Participants and Their Menstrual Cycles

Items	***n*** = 36 Persons	***n*** = 43 Cycles
Age: years, mean (SD)	27.7 (4.9)	
Weight: kg, mean (SD)	52.6 (4.6)	
BMI: mean (SD)	20.7 (1.3)	
Menstruation information		
Menstrual cycle: days, mean (SD)	29.6 (2.3)^a^	30.0 (3.3)^b^
Self-manageable symptoms during menstruation: number of persons (%)	22 (61.1)	25 (58.1)
Self-manageable symptoms before menstruation: number of persons (%)	17 (47.2)	21 (48.8)

^a^ Usual cycles reported by the participants.

^b^ Actual cycle length during the study.

BMI, body mass index; SD, standard deviation.

### Representative value and reference interval of UBP levels

UBP levels in all samples (*n* = 1260) were not normally distributed according to the Kolmogorov–Smirnov test (*p* = 0.000); thus, the median was adopted as a representative value, which was 0.2291 μmol/gCr. Non-normally distributed data were arranged in ascending order, and 95% of the data range was defined as the reference interval.^18,19^ The bottom and top 2.5% of data were set as the lower and upper limits, respectively. The 95% range of UBP levels was 0.0102–2.9335 μmol/gCr.

### Daily trend of UBP levels in follicular and luteal phases

The follicular phase occurs from the first day of menstruation to approximately the 14th day, the beginning of ovulation, although its length depends on the cycle.^20^ There was no significant correlation between daily UBP levels from the second day to the 14th day of menstruation (*n* = 550) and the order of the day according to Spearman's rank correlations (*ρ* = 0.017, *p* = 0.685). This analysis was also conducted for participants who had self-manageable symptoms during menstruation (*n* = 325), and no significant correlation was found (*ρ* = 0.041, *p* = 0.464).

The luteal phase lasts for 14 days before the start of menstruation.^20^ There was no correlation between daily UBP levels for 14 days from the last to reverse 14th day of menstruation (*n* = 584) and the reverse order of days (*ρ* = −0.049, *p* = 0.237). Similarly, correlations were explored for participants who had self-manageable symptoms before menstruation (*n* = 286); no significant correlation was found (*ρ* = 0.035, *p* = 0.560).

### Five-day cluster trend of UBP levels in follicular and luteal phases

**FIG. 2.**
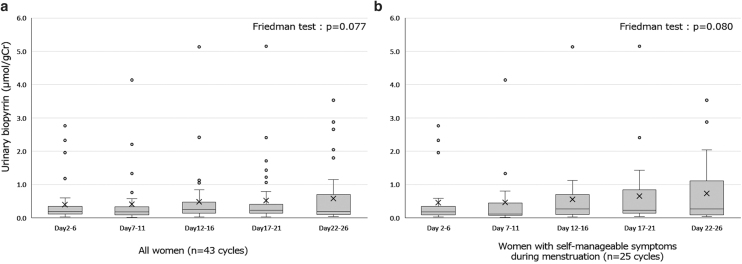
**(a)** Median UBP levels every 5 days from the second day of menstruation in all women (*n* = 43 cycles). **(b)** Median UBP levels every 5 days from the second day of menstruation in women with self-manageable symptoms during menstruation (*n* = 25 cycles).

The median UBP levels in each 5-day cluster from the second to 26th day of menstruation were compared using the Friedman test. There was no significant difference among the five groups (*p* = 0.077) (Fig. 2a). The same analysis was conducted for the 25 cycles of women with self-manageable symptoms during menstruation. The Friedman test indicated no significant difference in median UBP levels among the groups (*p* = 0.080) (Fig. 2b).

**FIG. 3.**
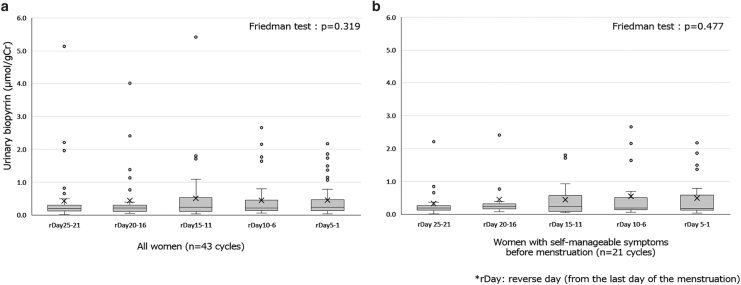
**(a)** Median UBP levels every 5 days from the last day of menstruation in all women (*n* = 43 cycles). **(b)** Median UBP levels every 5 days from the last day of menstruation in women with self-manageable symptoms before menstruation (*n* = 21 cycles).

In addition, median UBP levels among each 5-day cluster from the last day to the 25th day of menstruation in reverse order were analyzed using the Friedman test. No significant difference was identified among the five groups (*p* = 0.319) (Fig. 3a). Furthermore, the median UBP levels of 21 cycles of women with self-manageable premenstrual symptoms were extracted and compared among the five groups from the 5-day clusters. The Friedman test showed no significant difference in median UBP levels among the groups (*p* = 0.477) (Fig. 3b).

The Mann–Whitney *U* test revealed no significant difference in median UBP levels in the 5-day clusters from the second to 26th day of menstruation in participants with self-manageable symptoms during menstruation and those without symptoms (Table 2). The same analysis was conducted on median UBP levels in the 5-day cluster from the last day to the 25th day of menstruation, in reverse order, for participants who had self-manageable premenstrual symptoms and those who had no symptoms; there were no significant differences between the groups (Table 2).

**Table 2.** Median Urinary Biopyrrin Levels Every 5 Days With or Without Self-Manageable Symptoms During and Before Menstruation

Symptom(s)	Yes/No	Day 2–6	Day 7–11	Day 12–16	Day 17–21	Day 22–26
During menstruation	Yes (*n* = 25 cycles)	0.7348	0.8532	1.0069	1.0942	0.9330
	No (*n* = 18 cycles)	0.2360	0.2244	0.2316	0.2351	0.1710
	*p*	0.571	0.491	0.825	0.768	0.375

Symptom(s)	Yes/No	rDay 25–21	rDay 20–16	rDay 15–11	rDay 10–6	rDay 5–1
Before menstruation	Yes (*n* = 21 cycles)	0.1846	0.2397	0.2423	0.1794	0.1740
	No (*n* = 22 cycles)	0.2170	0.1956	0.2419	0.2455	0.3008
	*p*	0.356	0.827	0.576	0.264	0.680

Data were analyzed using the Mann–Whitney *U* test. To analyze data of participants with symptoms before menstruation, the days were counted in reverse from the last day of menstruation.

rDay, reverse day.

## Discussion

### Representative value of UBP

Since UBP levels in all samples were not normally distributed, the median was adopted as a representative value among healthy nonpregnant women, which was 0.2291 μmol/gCr (*n* = 1260). The mean UBP levels with one-spot urine from previous studies were 1.7 (0.9) μmol/gCr involving 35 nonpregnant women^14^ and 1.21 (0.61) μmol/gCr involving 40 healthy nonpregnant volunteers (including five males).^16^ Considering the large sample size and data distribution, the median calculated in our study could represent UBP levels of healthy nonpregnant women with greater reliability than those reported in previous studies.

Furthermore, our study presented the reference interval of the UBP level (0.0102–2.9335 μmol/gCr), which could only be measured with a large number of samples. The upper limit can be used as a potential indicator to assess values that require careful attention. The mean UBP levels reported by two previous studies^14,16^ were within this range.

### UBP levels in relation to the menstrual cycle

Daily median UBP levels were not significantly correlated with the order of days in the follicular phase or the reverse order of days in the luteal phase, indicating that there was no daily trend in UBP levels during the menstrual cycle. The median UBP level of each 5-day cluster also showed no significant difference, suggesting that even the obvious phenomena relevant to hormonal changes did not affect the UBP level reflecting oxidative stress; for example, menstrual bleeding (in the first cluster, day 2–6), the ovulatory phase with a luteinizing hormone surge (in the third cluster, day 12–16), and the mid-luteal phase with peak progesterone (in the second cluster beginning on the last day of menstruation, reverse day 6–10).^21^

Some studies support our findings. Tada et al.^16^ noted that UBP levels may be associated with school-related stress, whereas menstruation did not affect the urinalysis results. Wilson et al.^22^ reported that menstrual schedules in healthy women did not affect systematic shifts in salivary, urinary, and plasma cortisol levels.

Browne et al.^23^ measured biomarkers of oxidative stress, including lipid peroxidation, antioxidant enzymes, and antioxidant vitamins, in nine healthy women aged 18–44 years with regular menstrual cycles. No statistically significant differences or time effects were observed during menstrual cycles.

Konishi et al.^24^ also reported that there was no relationship between urinary concentration of 8-hydroxy-2′-deoxyguanosine (8-OHdG) and the menstrual cycle. Because oxidative stress during the menstrual cycle is reduced mainly by glutathione, which plays an important role in the intracellular antioxidant defense system and prevents cell damage caused by oxidative stress, the menstrual cycle in healthy nonpregnant women may have no significant relationship with daily UBP levels.

Ishikawa et al.^25^ found no significant differences in serum derivatives of reactive oxygen metabolite levels and biological antioxidant potential values between women in the early follicular phases and those in the mid-luteal phase, suggesting that estrogen fluctuations during the menstrual cycle may not affect oxidative status.

On the other hand, Karowicz-Bilinska et al.^26^ reported that higher levels of both indices of oxidative stress, urinary hydrogen peroxide and thiobarbituric acid-reactive substances, were found in the luteal phase of the menstrual cycle. Furthermore, Cornelli et al.^27^ indicated that healthy eumenorrheic women experience oxidative stress for two-thirds of the menstrual cycle.

Finally, the reasons for discrepancies in the relationships between oxidative stress indices and menstrual cycles remain unclear. Therefore, it is necessary to further investigate the correlation between them, focusing on sex hormones that fluctuate during the menstrual cycle.

### UBP in relation to symptoms during the menstrual cycle

The median UBP levels in the 5-day clusters in participants who had self-manageable symptoms during or before menstruation did not show any significant differences. These median UBP levels also did not show any differences compared with those in women who did not have symptoms.

The findings in our study may be attributed to the inclusion of women with self-manageable symptoms. Patients with more severe symptoms requiring gynecological treatment may have shown different results. In a previous study involving healthy women and patients with premenstrual syndrome,^28^ plasma 8-OHdG concentrations during the premenstrual phase were significantly higher than those during the postmenstrual period. This discrepancy supports the notion that biopyrrins and 8-OHdG reflect different oxidative stress levels.^11^

Iida et al.^29^ reported that the menstrual cycle did not show a significant correlation with urinary 8-OHdG levels, whereas the menstrual cycle-adjusted 8-OHdG level was significantly higher in participants with depressive symptoms than in those without symptoms. Further studies on UBP in women with symptoms of depression are warranted.

### Implications for clinical practice and limitations

It is well known that physical and psychological stresses are significantly complex and involve various responses. In addition, there are huge individual differences in the feelings and tolerance of stress; therefore, determining the baseline of stress for specific populations such as our study population would be critical for related research.

As this study indicated no significant relationship between UBP levels and the menstrual cycle, oxidative stress in healthy nonpregnant women can be evaluated using UBP without considering the menstrual cycle. UBP serves as a convenient marker for oxidative stress because urine can be collected noninvasively. Moreover, assessing UBP levels might help prevent possible overstress that may lead to psychosis and guide people to seek prompt medical attention when necessary.

This study had some limitations. First, the UBP assessments were performed under regular conditions by counting the days without clear detection of follicular and luteal phases with female hormone levels. Second, there were no stimulatory measures to determine the clinical response or its relationship with increased UBP levels. Therefore, further studies are required to apply UBP as an indicator of therapeutic response to treatment in stressed individuals. Finally, this study did not observe any relationship between UBP and other stress markers, such as cortisol or adrenaline.

Combined with several other markers, UBP can help monitor the mental status of people more comprehensively.

## Conclusions

This study demonstrated the median UBP level in healthy nonpregnant women as a representative value. The reference interval of the UBP level was also calculated using a large sample size, indicating that the upper limit can be used as a reference for overstress. Our findings suggest that UBP could be an objective oxidative stress indicator that is less sensitive to the menstrual cycle and concomitant symptoms.

UBP levels in healthy nonpregnant women can be assessed regardless of the menstrual cycle and concomitant symptoms.

## Abbreviations Used

8-OHdG: 8-hydroxy-2'-deoxyguanosine
BMI: body mass index
SD: standard deviation
UBP: urinary biopyrrin
